# Protease inhibitor associated mutations compromise the efficacy of therapy in human immunodeficiency virus – 1 (HIV-1) infected pediatric patients: a cross-sectional study

**DOI:** 10.1186/1742-6405-4-15

**Published:** 2007-07-09

**Authors:** Amisha Malhotra, Sunanda Gaur, Patricia Whitley-Williams, Caitlin Loomis, Anna Petrova

**Affiliations:** 1 Department of Pediatrics, Division of Infectious Disease, University of Medicine and Dentistry of New Jersey (UMDNJ) – Robert Wood Johnson Medical School, New Brunswick, New Jersey 08903, USA; 2 Princeton University, Princeton, New Jersey-08542, USA

## Abstract

**Background:**

Although the introduction of combined therapy with reverse transcriptase and protease inhibitors has resulted in considerable decrease in HIV related mortality; it has also induced the development of multiple drug-resistant HIV-1 variants.

The few studies on HIV-1 mutagenesis in HIV infected children have not evaluated the impact of HIV-1 mutations on the clinical, virological and immunological presentation of HIV disease that is fundamental to optimizing the treatment regimens for these patients.

**Results:**

A cross sectional study was conducted to evaluate the impact of treatment regimens and resistance mutation patterns on the clinical, virological, and immunological presentation of HIV disease in 41 children (25 male and 16 female) at the Robert Wood Johnson Pediatric AIDS Program in New Brunswick, New Jersey. The study participants were symptomatic and had preceding treatment history with combined ARV regimens including protease inhibitors (PIs), nucleoside reverse transcriptase inhibitors (NRTIs) and non-nucleoside reverse transcriptase inhibitors (NNRTIs). Fifteen (36.6%) children were treated with NRTI+NNRTI+ PI, 6 (14.6%) with NRTI+NNRTIs, 13 (31.7%) with NRTI+PIs, and the remaining 7 (17.1%) received NRTIs only.

Combined ARV regimens did not significantly influence the incidence of NRTI and NNRTI associated mutations. The duration of ARV therapy and the child's age had no significant impact on the ARV related mutations. The clinico-immunological presentation of the HIV disease was not associated with ARV treatment regimens or number of resistance mutations. However, primary mutations in the protease (PR) gene increased the likelihood of plasma viral load (PVL) ≥ 10,000 copies/mL irrespective of the child's age, duration of ARV therapy, presence of NRTI and NNRTI mutation. Viremia ≥ 10,000 copies/mL was recorded in almost all the children with primary mutations in the PR region (n = 12/13, 92.3%) as compared with only 50.0% (n = 14/28) of HIV infected children without (PR-), P < 0.008. However, CD-4 T cells were not affected by the mutations in the PR gene of the HIV-1 isolates.

**Conclusion:**

Primary PR resistance mutations significantly increase the likelihood for high viral replication in pediatric patients with moderate/severe HIV-1 infection, which may affect the long-term clinical prognosis of the HIV infected children.

## Background

Antiretroviral drug resistance produced by drug associated mutations in specific regions of the HIV genome has been recognized as one major problem during the treatment of human immunodeficiency virus type 1 (HIV-1) infected patients [1,2]. It has been shown that antiretroviral therapy used in the presence of drug-resistant viruses may increase the risk of HIV-1 mutagenesis [2,3] and the expansion of resistant HIV mutants compromises the efficacy of the antiretroviral (ARV) therapy [4-6].

Numerous studies have described the association between multiple mutations and multidrug resistance in HIV-1 isolates in adults [3,7-10] but knowledge regarding this issue in the population of HIV infected children is limited. We previously reported the occurrence of mutations in the protease (PR) gene in a small group of HIV infected children treated with the protease inhibitor (PI) nelfinavir [11]. HIV-1 mutants have been isolated both from both, ARV treated and untreated HIV infected Brazilian children [12] Several studies demonstrated that the failure of ARV therapy [13] and clinical disease progression [14] in HIV infected children is associated with a high frequency of primary mutations in the reverse transcriptase (RT) gene.

Cognizance of the impact of resistance mutations on the subsequent response to ARV therapy is of importance in the optimization of treatment regimens, especially for HIV-1 infected pediatric patients. The few studies published on the subject of HIV mutagenesis in children do not address the prime question regarding the extent to which HIV-1 mutations impact the clinical, virological, and immunological parameters of HIV disease in the infected children.

We designed a cross-sectional study to assess the impact of the development and association of drug resistant mutations in the HIV-1 genome on the clinical, virological, and immunological presentation of HIV infection in children who were infected at birth.

## Methods

An Institutional Review Board approved study was conducted at the Robert Wood Johnson Medical School's Pediatric Infectious Disease Clinic between 1999 and 2004. A total number of 42 HIV infected children were enrolled, and 41 (97.6%) of those who completed the required virological, immunological and HIV genotype testing were included in the analysis.

Demographic and treatment related information (gender, age of study entry, length and ARV treatment regimens) was recorded for each subject. In order to provide more prognostic information, plasma viral load (PVL) and CD4+ T- lymphocyte counts were tested simultaneously [15,16] using HIV-1 RNA assay flow cytometry [17,18]. Resistance mutations in the HIV genomes, both the RT and the PR genes were examined in the plasma viral RNA by reverse transcription and relevant genome segment amplification (HIV GENOSURE™). The presence of resistance-related mutations was classified in accordance with the recommendations of the International AIDS Society-USA [19] and International Expert Panel on HIV Antiretroviral Drug Resistance [20], and the 2005 update by Johnson et al [21] regarding drug resistance mutations in HIV-1 was also used.

The mutations associated with protease inhibitors (PIs), nucleoside reverse transcriptase inhibitors (NRTIs) and non-nucleoside reverse transcriptase inhibitors (NNRTIs) as well as the individual point (single amino acid changes) mutations were analyzed. Individual drug specific resistance and cross-resistance to one or more drugs of the same class was also identified.

The collected clinical and immunologic data were categorized in accord with the classification system for HIV-infected children of less than 13 years of age [22] and adolescents [23]. PVL cutoff of 10,000 copies/mL for initiation of ARV therapy in adults and adolescents [24] was used to evaluate viremia level <10,000 copies/mL and ≥ 10,000 copies/mL.

Clinical, immunological, and virological data was statistically analyzed (Statistica 6.0, StatSoft, Tulsa, OK) with respect to the treatment regimens and resistance mutations. The Pearson chi-square or Fisher's exact tests were performed for categorical data and Mann-Whitney test or Analysis of Variance for continuous data. Odds ratio (OR) and 95% confidence interval (95%CI) was calculated for the association using results from the multivariate regression analysis. All the P values presented are two-tailed with significance <0.05

## Results

**Figure 1:**
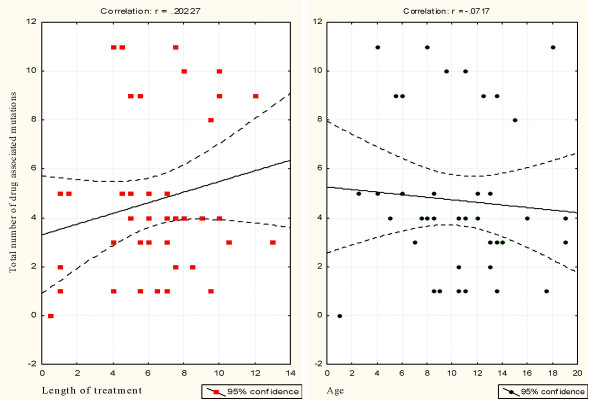
HIV-1 drug resistance mutations correlated with age and duration of ARV therapy (in years).

The 25 males and 16 females (n = 41) included in this study were moderately or severely symptomatic [22,23] at the time of study entry. The ARV treatment history showed that all but one had received two or more NRTI group medications (zidovudine, lamivudine, stavudine, didanosine, and zalcitabine). In addition, 21 (51.2%) children were treated with one or two medications from the NNRTI group (nevirapine, efavirenz) and 28 (68.3%) – with one or two medications from the PI group (nelfinavir, ritonavir, indinivir, saquinavir). However, PI boosted regimens were not used in the treatment of these patients. Overall, the majority of the HIV-1 infected children in this study (n = 34, 82.9%) had been treated with combined ARV regimens, such as (i) NRTI+NNRTI+PI (n = 15, 36.6%), (ii) NRTI+NNRTI (n = 6, 14.6%) and (iii) NRTI+PI (n = 13, 31.7%), and the rest (n = 7, 17.1%) received NRTIs only. Comparison of the average number of medications received by children from each ARV groups (Table 1) did not reveal any statistically significant differences. Moreover, we found no association between the ARV regimen, child's age and duration of the ARV treatment (Table 1). Variation in the clinical, immune and virological presentation of HIV disease and the mean number of mutations was not significantly associated. The total number of recorded HIV-1 isolate mutations did not correlate with length of therapy or the child's age (Figure 1).

**Table 1:** Demographic, clinical, and immunological data in association with the ARV treatment regimens

**Parameters**	**ARV treatment regimens**			
	
	**NRTI+NNRTI+PI** **(n = 15)**	**NRTI+NNRTI** **(n = 6)**	**NRTI + PI** **(n = 13)**	**NRTIs** **(n = 7)**
Age of study entry (Mean+/-SD, yrs)	11.9+/-4.3	10.8+/-3.4	8.8+/-5.1	10.4+/-3.0
Length of treatment (Mean+/-SD, yrs)	7.8+/-3.1	6.7+/-2.3	5.1+/-2.8	5.8+/-2.7
Number of Medications				
NRTI	3.7+/-0.7	3.3+/-1.0	3.1+/-1.0	2.7+/-1.1
NNRTI	1.1 +/-0.2	1.2+/-0.4	-	-
PI	1.1+/-0.6	-	1.2+/-0.4	-
Clinical categories *				
B	8 (53.3%)	3/5 (60.0%)	4 (30.8%)	3 (42.9%)
C	7 (46.7%)	2/5 (40.0%)	9(69.2%)	4 (57.1%)
Immune categories **				
1 (no suppression)	10 (66.3%)	4 (66.7%)	7 (53.9%)	3 (42.9%)
2–3 (moderate/severe)	7 (33.3%)	2 (33.3%)	6 (46.1%)	4 (57.1%)
PVL (n, %)				
<10,000	5 (33.3%)	4 (66.7%)	2 (15.4%)	4 (57.1%)
≥ 10,000	10 (66.7%)	2 (33.3%)	11 (84.6%)	3 (42.9%)
Number of mutations (Mean+/-SD)	5.7+/-3.8	3.5+/-1.6	4.7+/-3.2	3.7+/-2.3

*B-moderately, C-severely symptomatic ^22,23^

** Immune categories based on age-specific CD4+ T-lymphocyte count.^22,23^

**Figure 2:**
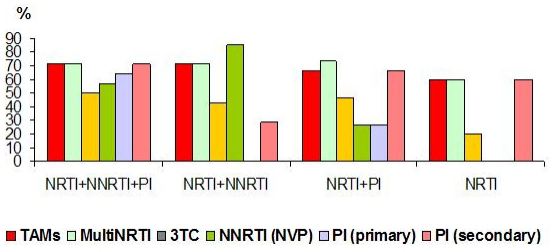
Presence of drug-specific HIV-1 mutations in relation to ARV regimens.

Individual analysis of the resistance mutations showed that HIV-1 isolates from all but one of the infected children treated with NRTI+PI regimens for 6 months developed different mutations in the RT and PR genes. Overall, drug associated mutations were recorded in HIV-1 isolates from 87.8% of children treated with NRTIs (n = 36/41), 66.7% treated with NNRTIs (n = 14/21), and 46.4% treated with PIs (n = 13/28), P < 0.03. As shown in Figure 2, combined ARV regimens did not significantly influence the incidence of NRTI and NNRTI associated mutations. The primary (I-II class) PI mutations in the HIV-1 isolates were twice as high in the children treated with NRTI+NNRTI+PI as compared with the isolates from children treated with NRTI+PI (60.0 % vs. 30.8%, P < 0.07). The duration of PI treatment in the children who had a history of ARV regimens including NRTI+ NNRTI+PI was an average of 45.1+/-24 months as compared with 29.9+/-19.2 months in those who were treated with NRTI+PI (P = 0.08). A few non-classified mutations in the HIV-1 genome such as F 214L (n = 1, 3.3%), I64V (n = 3, 10.0%), K10I (n = 3, 10.0%), R21I (n = 1, 3.3%), S68G (n = 2, 6.6%), and L214F (n = 1, 3.3%) were also observed in the children treated with PI medications. Moreover, the HIV-1 isolates from the NNRTI and PI naïve children showed NNRTI (n = 4/20, 20.0%) and secondary (class III-IV) PI associated mutations (n = 6/13, 46.1%).

As shown in Table 2, viremia ≥ 10,000 copies/mL was recorded in almost all children with primary mutations in the PR region (n = 12/13, 92.3%) as compared with only 50.0% (n = 14/28) of the HIV-1 infected children without the primary mutation (PR-), P < 0.008. Age-specific CD4 counts and immune categories were distributed with an almost similar frequency in the children with and without the PI associated mutations. No differences in age at study entry, length of previous ARV therapy and clinical stage of disease were observed with respect to the resistance mutations in the PR gene. We found that 3TC mutations and PI secondary mutations were more frequently seen in HIV-1 isolates with primary mutations in the PR gene. However, the presence of primary mutations in the PR gene (class I-II) significantly increased the likelihood of PVL ≥ 10,000 copies/mL irrespective of the child's age, duration of ARV therapy and presence of other mutations (Odds Ratio 1.7, 95% Confidence Interval 1.15, 2.52). At the same time, primary mutations in the PR gene did not significantly influence the severity of the clinical and immunological presentation of HIV disease in the studied children (Table 2). Moreover, apart from the PI associated mutations, there were no significant differences in the frequencies of the NRTI and NNRTI associated resistance mutations in the HIV-1 isolates in the children with PVL<10,000 copies/mL and >10,000 copies/mL (Figure 3).

**Table 2:** Demographic, clinical, and immunological data in association with the resistance mutations in the PR gene of the HIV-1 isolates

**Parameters**	**PI associated resistance mutations**	
	
	**PI (+)** **N = 13**	**PI (-)** **N = 28**
Age of study entry (Mean+/-SD, years)	10.2+/-5.2	10.6+/-2.7
Length of treatment (Mean+/-SD, years)	6.9+/-3.4	6.2+/-2.7
NNRTI therapy	9 (69.2%)	12 (42.9%)
TAMs mutations	10 (76.9%)	18 (64.3%)
3TC mutations	9 (64.2%) *	9 (32.1%)
NNRTI mutations	6 (46.1%)	12 (42.9%)
PI (secondary)	10 (76.9%) ***	15 (53.5%)
Clinical categories #		
B	6 (46.1)	12/27 (44.4%)
C	7 (53.9%)	15/27 (55.6%)
Immune categories ##		
1 (no suppression)	8 (61.5%)	16 (57.1%)
2–3 (moderate/severe)	5 (28.5%)	12 (42.9%)
PVL (n, %)		
<10,000	1 (7.7%) **	14 (50.0%)
≥ 10,000	12 (92.3%)	14 (50.0%)

Chi-square: * P < 0.02 ** P < 0.008 ***P < 0.0001

# Immune categories based on age-specific CD4+ T-lymphocyte count.^22,23^

## B-moderately, C-severely symptomatic ^22,23^

**Figure 3:**
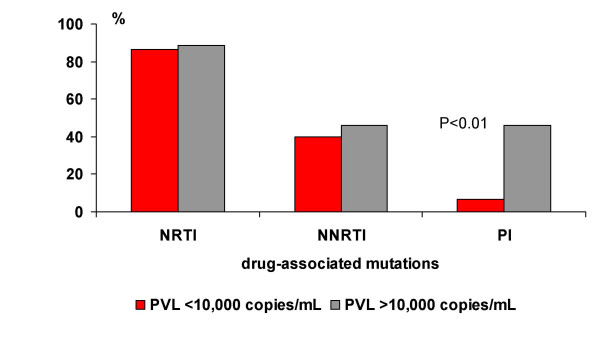
Association of drug-specific HIV-1 mutations and plasma viral load (PVL).

## Discussion

The introduction of combined therapy with reverse transcriptase and protease inhibitors has resulted in a considerable decrease in the HIV-1 related mortality [25]. Although the therapeutic regimens with the combined medications increase the selective pressure against viruses, they also subsequently induce the development of multiple drug-resistant HIV-1 variants [26]. The present study shows that NRTI -associated resistance mutations occur nearly always in HIV-1 infected pediatric patients receiving NRTIs, but NNRTI and PI-associated mutations are less likely to occur in children whose treatment includes NNRTIs and PIs (87.8% vs. 66.7% vs. 46.1%, respectively, P < 0.03). The rate of virus replication ≥ 10,000 copies/mL is significantly associated with the PI medication resistance mutations in the PR region at codons 10, 46, 54, 82 and 90 which are known to affect susceptibility to all currently FDA-approved PIs, and codon 30 (D30N), which may also significantly affect the virological response [27]. In contrast, four or more mutations are required to overcome the activity of PI medications in adults [28]. It is possible that the thymidine-associated mutations (TAMs) recorded in the HIV-1 infected children in the present study may mediate resistance to multiple mutations unrelated to the NRTI treatment [29,30]. The mutations associated with more than one NRTI may increase viral replication in an additive manner [2,31,32] and NNRTI associated mutations may intensify the rebound in the HIV-1 RNA [33]. The observed association between the higher PVL and resistance mutations in the PR region was confirmed after controlling for mutations in the RT regions. The persistent high-level of viral replication seen in children with primary mutations in the PR gene may increase the accumulation of multiple resistance mutations [34-38] and therefore affect the efficacy of drug therapy.

Although high-level virus production is associated with CD4+ T-cell destruction in adults [39], no such association was identified between viral replication and severity of immune suppression in the children included in the present study. Resino et al [40] identified the possibility of CD4+ T-cell recovery despite virological failure resulting in the discordant response in children. Moreover, viral load suppression is not indispensable for recovery of CD4+levels [41].

We have to acknowledge that insufficient drug potency, pharmacological issues, and poor compliance could be responsible for the anti-viral treatment failure [20], in addition to the drug resistance mutations in the PR region that were identified as a major predictor for the failure in the suppression of viral replication in this study. Moreover, despite the disagreement in the literature [42-44], medically directed treatment interruptions, which were not analyzed in this study, may influence the virologic and immunologic outcome as well as the development of drug-resistant viral isolates. Long-term clinical and immunologic follow up will be required in order to clarify the predictive power of HIV-1 RNA plasma levels in the monitoring of disease progression.

## Conclusion

Despite the limitations, this study clearly demonstrates that primary PR resistance mutations significantly increase the likelihood for high viral replication in children with moderate/severe HIV-1 infection. This raises concern regarding early failure in drug susceptibility, which may jeopardize the long-term clinical prognosis of the HIV-1 infected pediatric patient.

## Abbreviations

HIV-1, human immunodeficiency virus type 1; ARV, antiretroviral; PVL, plasma viral load; CD4+, T- lymphocyte count; NRTI, nucleoside reverse transcriptase inhibitor; NNRTI, non-nucleoside reverse transcriptase inhibitor; PI, protease inhibitor; PR, mutation in the protease gene; RT, reverse transcriptase; PR, protease; TAM, thymidine associated mutation.

## Competing interests

The author(s) declare that they have no competing interests.

## Authors' contributions

AM participated in study design and coordination, data collection and preparation of manuscript.  SG participated in study design and coordination.  PWW participated in study design and coordination.  CL carried out the data collection.  AP  participated in study design, statistical analysis, and preparation of manuscript.  All authors have read and approved the final manuscript.
